# The ncRNA-Mediated Overexpression of Ferroptosis-Related Gene EMC2 Correlates With Poor Prognosis and Tumor Immune Infiltration in Breast Cancer

**DOI:** 10.3389/fonc.2021.777037

**Published:** 2021-12-08

**Authors:** Xing Liu, Pengshuo Yang, Lu Han, Qing Zhou, Qingsong Qu, Xinyuan Shi

**Affiliations:** ^1^ School of Chinese Materia Medica, Beijing University of Chinese Medicine, Beijing, China; ^2^ School of Life Science, Beijing University of Chinese Medicine, Beijing, China

**Keywords:** ferroptosis-related gene, breast cancer, tumor immune cell infiltration, immune cell biomarkers, disease prognosis

## Abstract

Ferroptosis is an iron-dependent programmed cell death process. Although ferroptosis inducers hold promising potential in the treatment of breast cancer, the specific role and mechanism of the ferroptosis-related gene *EMC2* in breast cancer have not been entirely determined. The potential roles of *EMC2* in different tumors were explored based on The Cancer Genome Atlas (TCGA), Genotype-Tissue Expression (GTEx), Gene Expression Profiling Interactive Analysis 2 (GEPIA2), Tumor Immune Estimation Resource (TIMER), Shiny Methylation Analysis Resource Tool (SMART), starBase, and cBioPortal for cancer genomics (cBioPortal) datasets. The expression difference, mutation, survival, pathological stage, DNA methylation, non-coding RNAs (ncRNAs), and immune cell infiltration related to *EMC2* were analyzed. Gene Ontology (GO) and Kyoto Encyclopedia of Genes and Genomes (KEGG) analyses were performed to identify the differences in biological processes and functions among different related genes. The expression levels of core prognostic genes were then verified in breast invasive carcinoma samples using immunohistochemistry and breast invasive carcinoma cell lines using real-time polymerase chain reaction. High expression levels of *EMC2* were observed in most cancer types. *EMC2* expression in breast cancer tissue samples correlated with poor overall survival. *EMC2* was mutated and methylated in a variety of tumors and affected survival. The LINC00665-miR-410-3p axis was identified as the most potential upstream ncRNA-related pathway of *EMC2* in breast cancer. *EMC2* levels were significantly positively correlated with tumor immune cell infiltration, immune cell biomarkers, and immune checkpoint expression. Our study offers a comprehensive understanding of the oncogenic roles of *EMC2* across different tumors. The upregulation of *EMC2* expression mediated by ncRNAs is related to poor prognosis and tumor immune infiltration in breast cancer.

## Introduction

Cancer is the leading cause of death and an essential obstacle to increasing life expectancy, globally. With the aging of populations and increasing socio-economic development, the burden of cancer is rapidly increasing. According to the 2020 Global Cancer Statistics Report (1), female breast cancer had surpassed lung cancer to become the world’s most commonly diagnosed cancer, with an estimated 2.3 million new cases in 2020 (11.7%); it was also the fifth leading cause of cancer deaths worldwide, with 685,000 deaths. In China, the number of new cases of breast cancer ranks fourth, after those of lung cancer, colorectal cancer, and gastric cancer. Therefore, there is an urgent need to develop effective therapeutic targets or identify promising prognostic biomarkers for breast cancer.

The term “ferroptosis” was first proposed in 2012, and it has since become one of the research hotspots in the field of tumors. Ferroptosis is considerably different from other cell death types in terms of morphology, molecular biology, and metabolic characteristics. Furthermore, the activation of ferroptosis can inhibit the proliferation of tumor cells, which is speculated to become a new tumor treatment method (2). The destruction of the endoplasmic reticulum membrane protein complex (EMC) family affects several cellular processes, including protein transport, organelle communication, endoplasmic reticulum stress, virus maturation, and lipid homeostasis. The EMC is closely related to neurological diseases and tumors. Rare *EMC1* mutations are associated with severe human neurodegenerative diseases, which manifest as growth retardation, cerebellar atrophy, scoliosis, hypotonia, psychomotor retardation, epilepsy, and craniofacial abnormalities (3–5).

Changing the expression level of EMC subunits can regulate tumor growth in several human cancer models. *EMC6* knockdown increases the proliferation of glioblastoma cells, while its overexpression slows down cell proliferation, inhibits invasiveness, and enhances chemotherapy sensitivity (6). *EMC10-2* overexpression also inhibits glioma-induced cell cycle progression and invasion (7). In gastric cancer cells, *EMC6* overexpression reduces invasiveness and induces cell cycle arrest and apoptosis, but it does not induce autophagy (8). In a study on the corresponding target screening of ferroptosis, *EMC2*, also known as: *KIAA0103*, *TTC35*, was found more sensitive to erastin; however, there is no report on the function of this gene in tumor immunotherapy, which is an obstacle to clarifying the relationship between *EMC2* and tumor immunology.

Many factors regulate gene expression. Among them, ncRNAs are important regulators of eukaryotic gene expression and are related to monoallelic expression in other organisms (9). miRNA is a small endogenous ncRNA that negatively regulates the target gene expression (10). The long-chain gene expression in lncRNA regulatory factors play an important role in regulating many biological processes (11).

In this study, we performed expression, survival, and mutation analyses for *EMC2* in various human cancers; to the best of our knowledge, this is the first study on these lines. In addition, we discussed the regulation of non-coding RNA (ncRNA) related to *EMC2*, including microRNA (miRNA) and long non-coding RNA (lncRNA), in breast cancer. Finally, we determined the relationship between *EMC2* expression and immune cell infiltration, immune cell biomarkers, or immune checkpoints in breast invasive carcinoma (BRCA). Taken together, our findings indicate that the ncRNA-mediated upregulation of *EMC2* expression is associated with prognosis and poor tumor immune infiltration in of BRCA.

## Materials and Methods

### TCGA and Oncomine Data Retrieval and Analysis

TCGA tumor dataset for 33 cancer types was downloaded from https://genome-cancer.ucsc.edu/. For differential expression analysis, the R (version “4.1”) package ‘limma’ was used (12). Oncomine, a cancer microarray database and web-based data-mining platform aimed at facilitating discovery from genome-wide expression analyses (https://www.oncomine.org/resource/login.html). Search for “*TTC35*”, threshold (*p*-value): 0.05, threshold (fold change): 1.5, threshold (gene rank): Top 10%, data type: all (13). A *p*-value < 0.05 was considered statistically significant.

### GEPIA2 Database Analysis

Gene expression profiling interactive analysis 2 (GEPIA2; http://gepia2.cancer-pku.cn/#index/) is a tumor/normal differential expression analysis based on TCGA and gene tissue expression (GTEx) data, for analysis according to cancer type or pathological stage, patient survival analysis, network tools for similar gene detection, correlation analysis, and dimensionality reduction analysis (14). GEPIA2 was used to detect the expression of *EMC2* and lncRNA in different types of human cancers, with *p* < 0.05 considered statistically significant. It was also used to analyze the survival of *EMC2* in 33 different cancer types (including overall survival [OS] and relapse-free survival [RFS]), as well as to evaluate the prognostic value of candidate lncRNAs in BRCA. A log-rank *p*-value < 0.05 was considered statistically significant. In addition, the GEPIA2 database was used to evaluate the correlation between *EMC2* expression in BRCA and immune checkpoints. Absolute values of R > 0.1 and *p* < 0.05 were judged as statistically significant. Univariate and multivariate Cox proportional hazards models were used for survival analysis, and the R package ‘Survival’ (2.41-3) was used for the Kaplan–Meier estimation of OS.

### *EMC2* Mutation Analysis

After logging in to the cBioPortal website (https://www.cbioportal.org/), “TCGA Pan-Cancer Atlas Research” was selected in the “Quick Selection” column, and “*EMC2*” was entered to query gene mutation characteristics (15). We observed the mutation frequency, mutation type, and copy number variation (CNV) results of all TCGA tumors in the “Tumor Type Summary” module, and observed the mutation site information of *EMC2* in the protein structure diagram through the “mutation” module. We also used the “comparison” module to obtain data on overall, disease-free, progression-free, and disease-free survival differences in TCGA cancer cases with or without *EMC2* genetic changes. Kaplan–Meier plots of log-rank *p*-values were also generated.

### *EMC2* CpG Site Methylation Analysis

Shiny methylation analysis resource tool (SMART; http://www.bioinfo-zs.com/smartapp/) is a user-friendly and easy-to-use web application for comprehensively analyzing the DNA methylation data of TCGA (16). Here, by searching “*EMC2*,” we explored the difference in the average methylation level of the tumor and normal groups of *EMC2* in each type of cancer and the effect of methylation on tumor survival.

### Candidate miRNA Prediction

Several target gene prediction programs, such as PitA, RNA22, miRmap, microT, Miranda, PicTar, and TargetScan, were used to predict the upstream binding miRNAs of *EMC2* (17). Only predicted miRNAs usually appear in the above two programs before they are used in subsequent analyses. These predicted miRNAs were considered candidate miRNAs for *EMC2*.

### StarBase Analysis

starBase (http://starbase.sysu.edu.cn/) is the most comprehensive database of mRNA and protein-RNA interaction networks (18). We used starBase to analyze the expression correlation of miRNA-*EMC2*, hsa-miR-410-3p-lncRNA, or lncRNA-*EMC2* in BRCA. TCGA data was used to analyze the expression levels of hsa-miR-410-3p and lncRNA in BRCA and normal controls.

### Kaplan–Meier Plotter Analysis

The online Kaplan–Meier plotter (http://kmplot.com/analysis/) was used to perform a hsa-miR-410-3p survival analysis on BRCA, and a log-rank *p*-value < 0.05 was considered statistically significant (19).

### TIMER Database Analysis

The tumor immune estimation resource (TIMER, https://cistrome.shinyapps.io/timer/) is a web server for the comprehensive analysis of tumor-infiltrating immune cells (20). TIMER is used to analyze the correlation between the expression level of *EMC2* in BRCA and the expression level of immune checkpoints, and used the R package ‘GSVA’ to analyze the relationship between the expression level of *EMC2* and immune cell infiltration in breast cancer. *p* < 0.05 was considered statistically significant.

### Cell Culture

The human breast epithelial cell line MCF10A and human breast cancer cell lines MCF-7 and MDA-MB-231 were obtained from Bei Na Biotechnology Co., Ltd. (Beijing, China). The MCF7 and MDA-MB-231 cells were maintained in high-glucose Dulbecco’s modified Eagle medium (DMEM; Gibco, Grand Island, NY, USA) supplemented with 10% fetal bovine serum (FBS; Gibco), and 1% penicillin–streptomycin (Gibco). The MFC10A cells were cultured in DMEM/Ham’s F-12 medium (Invitrogen) supplemented with 10% FBS (Gibco), 0.5 µg/ml hydrocortisone (Sigma-Aldrich, St. Louis, MO, USA), 20 ng/ml epidermal growth factor (Sigma-Aldrich), 10 μg/ml insulin (Sigma-Aldrich), and 1% penicillin–streptomycin (Gibco). Cells were cultured in a humidified atmosphere containing 5% CO_2_ and 95% air at 37°C.

### *EMC2* Expression Analysis

Total RNA was extracted from the normal human breast epithelial cell line MCF 10A and breast cancer cell lines MCF7 and MDA-MB-231 using TRIzol reagent (Sigma-Aldrich). The extracted RNA was reverse-transcribed to cDNA using HiScript II Q RT SuperMix (R223-01; Vazyme Biotech, Nanjing, China). Real-time PCR was performed on a QuantStudio™ 6 Flex (Life Technologies, USA) using ChamQ SYBR qPCR Master Mix (Q711-02; Vazyme Biotech, Nanjing, China). The 2^–ΔΔCt^ method was used to calculate the relative gene expression levels and then normalized against *GAPDH*. Primers were designed and synthesized by Sangon Biotech (Shanghai, China). The primer sequences are listed in **Supplementary Table 1**.

### Statistical Analysis

The statistical analysis in this study was automatically calculated by the online databases mentioned in the above sections. A *p*-value < 0.05 or log-rank *p*-value < 0.05 was considered to be statistically significant.

## Results

### Differences in the Expression of *EMC2* in Tumors

**Figure 1:**
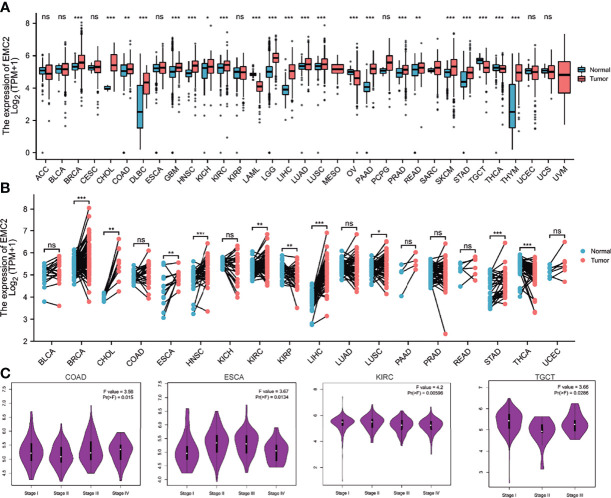
EMC2 expression analysis in multiple cancers. **(A)** The expression of EMC2 in 33 types of human cancer based on the TCGA (cancer samples) and GTEx (normal tissues of unpaired samples) databases. **(B)** EMC2 expression in paired samples obtained from TCGA. **(C)** Based on the TCGA data, the expression levels of the EMC2 were analyzed by the main pathological stages (stage I, stage II, stage III, and stage IV) of COAD, ESCA, KIRC, and TGCT. Log2 (TPM + 1) was applied for log-scale. ns, not significant; *P < 0.05; **P < 0.01; ***P < 0.001.

To explore the possible role of *EMC2* in carcinogenesis, we first analyzed its expression in 33 human cancers. As shown in **Figure 1A**, compared with the normal group, *EMC2* was expressed differently in 22 cancer types, of which 18 were highly expressed in the tumor group, that included breast invasive carcinoma (BRCA), cholangiocarcinoma (CHOL), colon adenocarcinoma (COAD), lymphoid neoplasm diffuse large B-cell lymphoma (DLBC), glioblastoma multiforme (GBM), head and neck squamous cell carcinoma (HNSC), kidney chromophobe (KICH), kidney renal clear cell carcinoma (KIRC), brain lower grade glioma (LGG), liver hepatocellular carcinoma (LIHC), lung adenocarcinoma (LUAD), lung squamous cell carcinoma (LUSC), pancreatic adenocarcinoma (PAAD), prostate adenocarcinoma (PRAD), rectum adenocarcinoma (READ), stomach adenocarcinoma (STAD), skin cutaneous melanoma (SKCM), and thymoma (THYM). There were four cancer types with low expression in the tumor group, comprising acute myeloid leukemia (LAML), ovarian serous cystadenocarcinoma (OV), testicular germ cell tumors (TGCT), and thyroid carcinoma (THCA). However, BRCA, CHOL, DLBC, GBM, HNSC, KIRC, LAML, LIHC, LUAD, LUSC, OV, PAAD, PRAD, SKCM, STAD, TGCT, and THYM showed significant differences in 17 tumors. We further verified the expression of *EMC2* in tumors containing paired samples. As shown in **Figure 1B**, there were significant differences in the expression levels of *EMC2* in BRCA, CHOL, esophageal carcinoma (ESCA), HNSC, KIRC, papillary renal cell carcinoma (KIRP), LIHC, LUSC, STAD, and THCA compared with the corresponding adjacent normal tissue controls. Second, we used GEPIA2 to analyze the influence of *EMC2* expression on tumor grading and found significant differences in COAD, ESCA, KIRC, and TCGT (**Figure 1C**). The Oncomine database was used to evaluate the differential expression of *EMC2* in tumors, and similar results were obtained (**Supplementary Figure 1**). The above results show that *EMC2* is a potential oncogene for eight cancers.

### *EMC2* Expression Affects the Prognosis of Human Cancer

**Figure 2:**
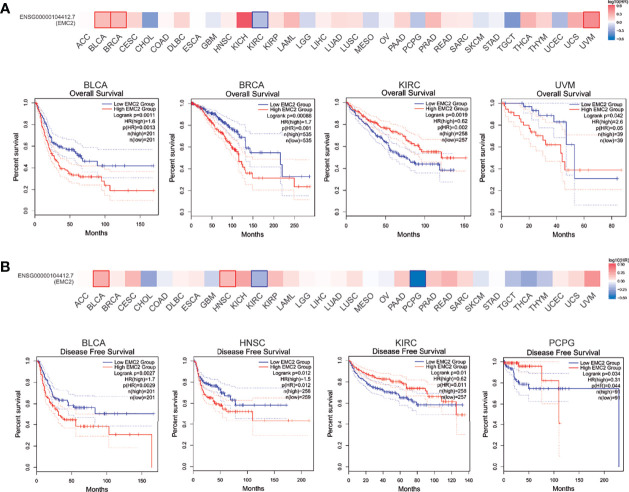
Correlation between *EMC2* gene expression and survival prognosis of cancers in TCGA. We used the GEPIA2 tool to perform overall survival **(A)** and disease free survival **(B)** analyses of different tumors in TCGA by *EMC2* gene expression. The survival map and Kaplan-Meier curves with positive results are given.

Next, a pan-cancer survival analysis was performed; it included two prognostic indices: OS and RFS. For OS, a high *EMC2* expression level showed unfavorable prognoses in bladder urothelial carcinoma (BLCA), BRCA, and uveal melanoma (UVM); however, KIRC patients with higher *EMC2* expression showed a better prognosis (**Figure 2A**). For RFS, BLCA and HNSC patients with high *EMC2* expression levels showed poor prognoses, while KIRC and pheochromocytoma and paraganglioma (PCPG) patients with high *EMC2* expression levels had better prognostic survival (**Figure 2B**). On this basis, we used univariate and multivariate Cox regression to analyze the effect of *EMC2* expression and other clinicopathological factors on breast cancer prognosis. The top 300 positive and negative correlation genes of *EMC2* were then subjected to GO and KEGG enrichment analyses, respectively. As shown in **Supplementary Table 2**, the results show that *EMC2* expression had an important effect on breast cancer and could be used as an independent prognostic indicator; these findings were irrespective of the regression analysis used (univariate or multivariate). Therefore, the expression of *EMC2* in breast cancer can have significant effects on factors such as tumor progression and even patient survival. GO and KEGG enrichment analysis found that genes positively related to *EMC2* are mainly involved in RNA transport and cell cycle regulation (**Supplementary Figure 2**), whereas genes negatively related to *EMC2* are mainly involved in ECM-receptor interaction, the relaxin signaling pathway, and focal adhesion (**Supplementary Figure 3**).

### Effect of *EMC2* Mutations on Human Cancer

**Figure 3:**
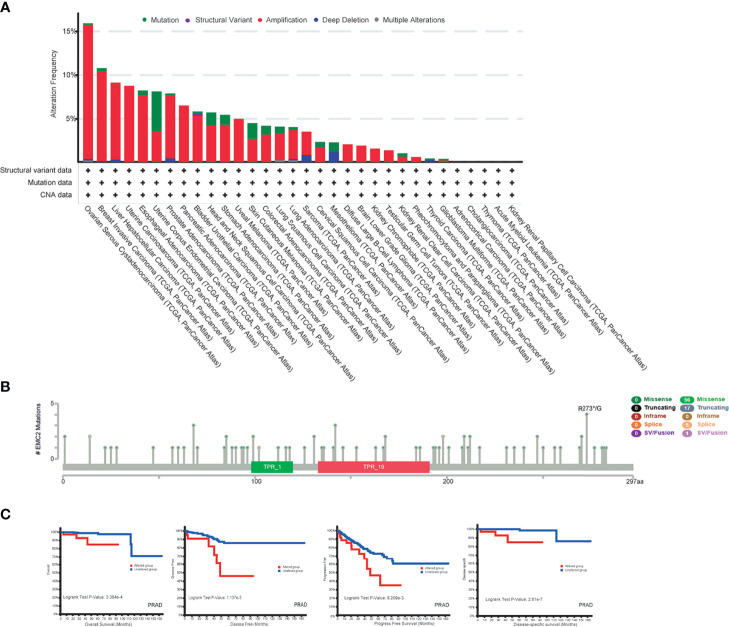
Mutation feature of *EMC2* in different tumors of TCGA. **(A)** We analyzed the mutation features of *EMC2* for the TCGA tumors using the cBioPortal tool. **(B)** We also analyzed the potential correlation between mutation status and overall, disease-specific, disease-free and progression-free survival of LIHC **(C)** using the cBioPortal tool.

We observed *EMC2* mutations in different tumor samples in TCGA cohort. As shown in **Figure 3A**, the frequency of *EMC2* mutations in OV patients with “amplification” as the primary type was the highest (>15.41%), followed by breast cancer (10.33%). Uterine corpus endometrial carcinoma (UCEC) was mainly of the “mutation” type, and its mutation frequency was about 4.54%. Some patients (about 1.15%) with mesothelioma lacked *EMC2*. **Figure 3B** shows the types and locations of *EMC2* mutations. In addition, we explored the potential relationship between *EMC2* mutations and the clinical survival and prognosis among patients with different cancer types. **Figure 3C** shows that compared with PRAD patients without *EMC2* mutations, those with *EMC2* mutations had decreased OS (*p* = 3.384e-4), disease-free survival (*p* = 1.137e-3), and progression-free survival (*p* = 8.209e-4), whereas the disease-specific survival rate (*p* = 2.81e-7) was particularly poor. In breast cancer patients, survival was not affected by the above mentioned mutations. Considering the number of clinical samples, the effect of *EMC2* structural mutations on breast cancer prognosis requires further analysis.

### Effect of *EMC2* CpG Site Methylation on Human Cancer

**Figure 4:**
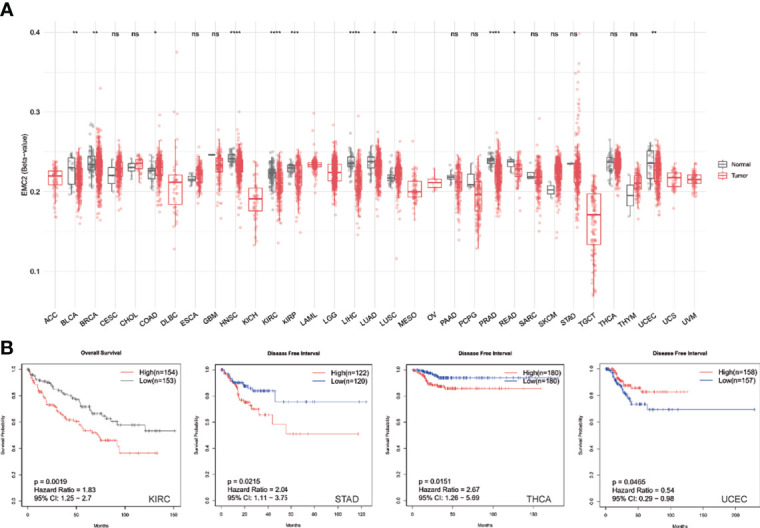
Pan-cancer EMC2 methylation analysis. **(A)** Pan-cancer differential expression of EMC2. **(B)** The impact of EMC2 methylation on survival. ns, not significant; *P < 0.05; **P < 0.01; ***P < 0.001; ****P < 0.0001.

DNA methylation can function to silence tumor suppressor genes along with genetic mutations. Thus, we calculated differentially methylated CpG sites based on the tumor and normal samples. The CpG sites of the *EMC2* promoter were significantly methylated in the tumor samples compared to in the normal samples of most cancer types (**Figure 4A**). Moreover, *EMC2* methylation had a significant effect on the survival of tumors (**Figure 4B**). We found that *EMC2* showed hypomethylation in BLCA, BRCA, HNSC, KIRC, KIRP, LIHC, LUAD, PRAD, READ, and UCEC, but hypermethylation in COAD and LUSC.

### Prediction and Analysis of miRNA Upstream of *EMC2*

**Figure 5:**
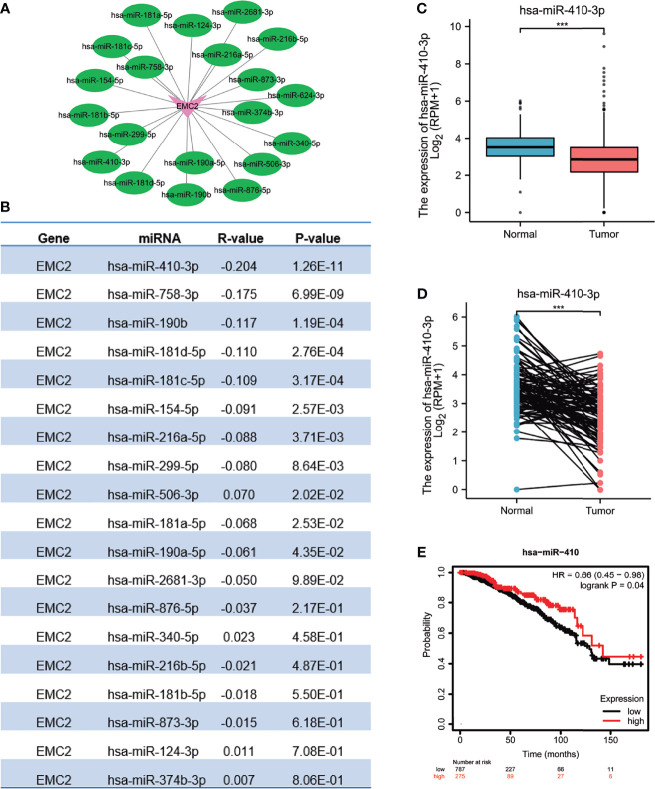
Identification of hsa-miR-410-3p as a potential upstream miRNA regulating EMC2 expression in BRCA. (**A**) The miRNA-EMC2 regulatory network established using Cytoscape software. (**B**) The expression correlation between predicted miRNAs and EMC2 in BRCA analyzed using starBase database. (**C**) hsa-miR-410-3p expression in breast cancer based on TCGA (cancer tissue) and GTEx (normal tissues of unpaired samples) databases. (**D**) hsa-miR-410-3p expression in breast cancer based on paired cancer samples obtained from TCGA. (**E**) The prognostic value of hsa-miR-410-3p in BRCA assessed by Kaplan-Meier plotter. ***P < 0.001.

To determine whether ncRNAs regulate *EMC2*, we first predicted upstream miRNAs that could bind to *EMC2*, and found 19 such miRNAs. To improve visualization, a miRNA–*EMC2* regulatory network was established using Cytoscape software (**Figure 5A**). Based on the mechanism of miRNA in regulating the target gene expression, we speculated a negative correlation between miRNA and *EMC2*; therefore, an expression correlation analysis was performed. As shown in **Figure 5B**, *EMC2* expression was significantly negatively correlated with miR-410-3p expression. The expression and prognostic value of miR-410-3p in BRCA were also determined. As mentioned above, in **Figures 5C, D**, miR-410-3p expression was significantly downregulated in BRCA, and its upregulated expression was positively correlated with prognosis (**Figure 5E**). These findings could that miR-410-3p is the most significant miRNA regulating *EMC2* in BRCA.

### Prediction and Analysis of lncRNA Upstream of miR-410-3p

**Figure 6:**
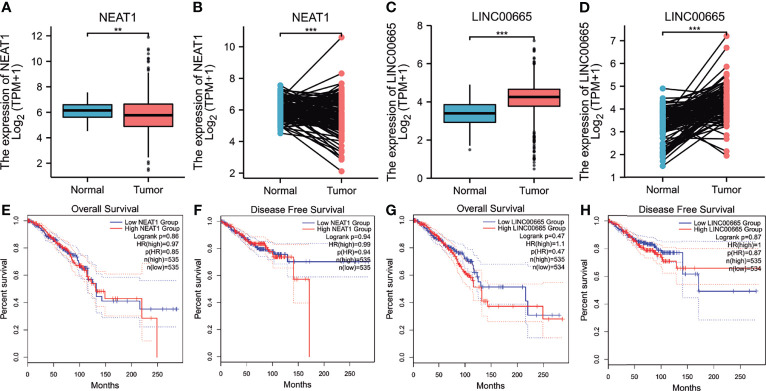
Expression analysis and survival analysis for upstream lncRNAs of hsa-miR-410-3p in BRCA. **(A–D)** The expression of NEAT1 **(A, B)** and linc00665 **(C, D)** in TCGA BRCA samples compared with “TCGA and GTEx normal tissues of unpaired samples” or “TCGA paired samples” data. **(E–H)** The OS analysis for NEAT1 **(E)** and LINC00665 **(G)** in BRCA. The RFS for NEAT1 **(F)** and LINC00665 **(H)** in BRCA. *p value < 0.05. **P < 0.01; ***P < 0.001.

Using starBase, 30 lncRNAs were predicted that were upstream of miR-410-3p. Similarly, to improve visualization, the lncRNA-miR-410-3p regulatory network was constructed using Cytoscape software (**Supplementary Figure 4**). Then, the corresponding normal tissue data in BRCA were extracted using GTEx and TCGA to determine the expression levels of these lncRNAs in BRCA. As shown in **Figures 6A–D**, among all the 30 lncRNAs, only NEAT1 and LINC00665 were significantly down- and upregulated in BRCA, respectively, compared to the normal control. Subsequently, the prognostic value of the two lncRNAs in BRCA was evaluated. As shown in **Figures 6E–H**, these lncRNAs did not significantly influence the survival of breast cancer patients. According to the competing endogenous RNA (ceRNA) hypothesis, lncRNA can increase mRNA expression by competitively binding and sharing miRNA. Therefore, a negative correlation between lncRNA and miRNA or a positive correlation between lncRNA and mRNA plausibly exists. As shown in **Table 1**, the starBase database was used to detect the expression correlation between the two lncRNAs in BRCA and miR-410-3p or *EMC2*. Using the expression analysis, survival analysis, and considering the correlation analysis, LINC0065 was found the most significant lncRNA upstream of the miR-410-3p/*EMC2* axis in BRCA.

**Table 1:** Correlation analysis between lncRNA and miRNA-410-3p or lncRNA and EMC2 in BRCA determined by starBase database.

lncRNA	miRNA	R-value	*p* value
NEAT1	miRNA-410-3p	0.111	2.54E-04
LINC00665	miRNA-410-3p	-0.112	2.12E-04
**lncRNA**	**mRNA**	**R-value**	***p* value**
NEAT1	EMC2	-0.071	1.89E-02
LINC00665	EMC2	0.172	9.42E-09

### Correlation Between *EMC2* Expression and Immune Cell Infiltration in BRCA

**Figure 7:**
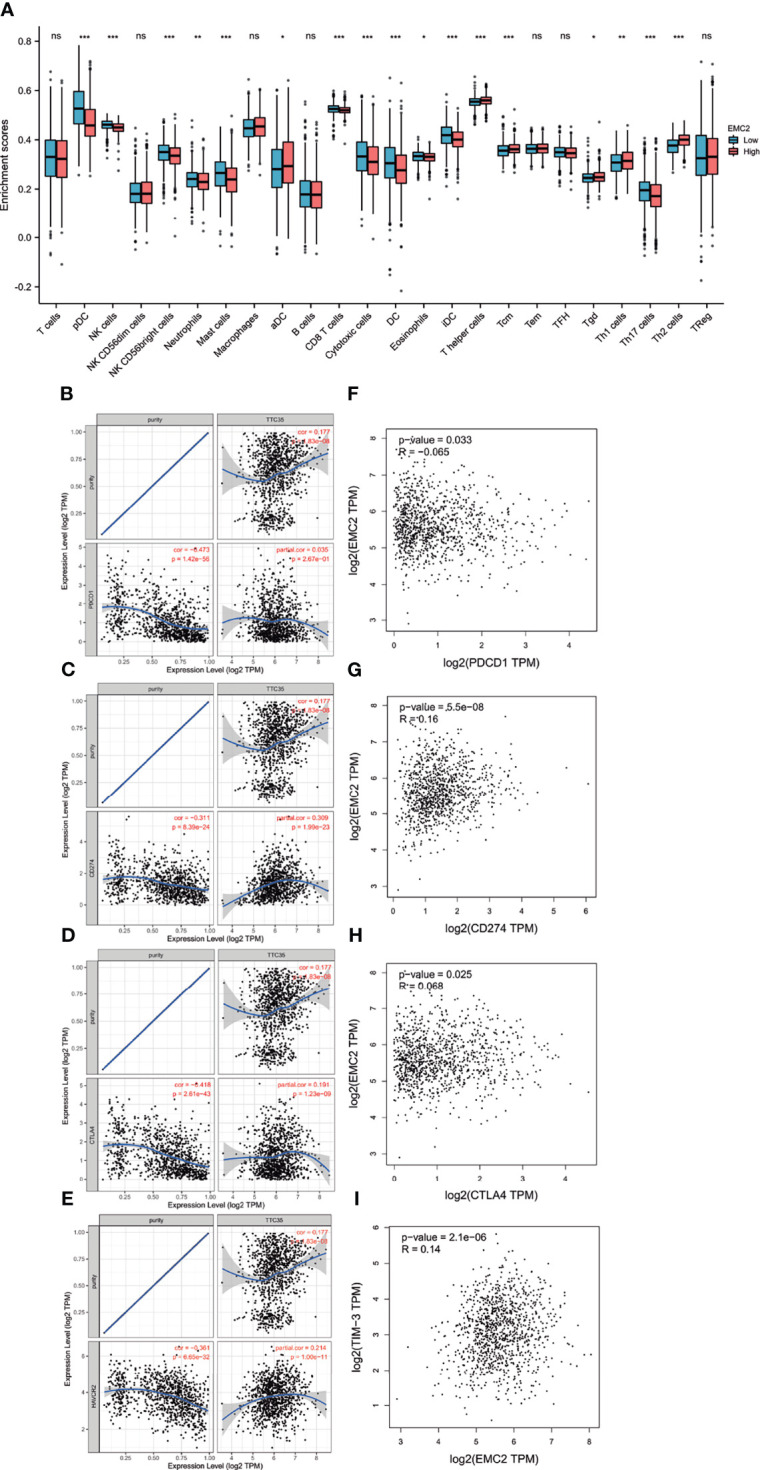
The relationship of immune cell infiltration with EMC2 expression and correlation of EMC2 expression with PD-1, PD-L1, CTLA-4 and TIM-3 expression in BRCA. **(A)** Correlation of EMC2 expression with the infiltration of different immune cells in BRCA. **(B)** Spearman correlation of EMC2 with expression of PD-1 in BRCA adjusted by purity using TIMER. **(C)** Spearman correlation of EMC2 with expression of PD-L1 in BRCA adjusted by purity using TIMER. **(D)** Spearman correlation of EMC2 with expression of CTLA-4 in BRCA adjusted by purity using TIMER. **(E)** The expression correlation of EMC2 with TIM-3 in BRCA adjusted by purity using TIMER. **(F)** The expression correlation of EMC2 with PD-1 in BRCA determined by GEPIA database. **(G)** The expression correlation of EMC2 with PD-L1 in BRCA determined by GEPIA database. **(H)** The expression correlation of EMC2 with CTLA-4 in BRCA determined by GEPIA database. **(I)** The expression correlation of EMC2 with TIM-1 in BRCA determined by GEPIA database. ns, not; significant; *P < 0.05; **P < 0.01; ***P < 0.001.

Immunotherapy is a treatment method that enhances or inhibits immune system function to cure diseases. Immunotherapy has achieved satisfactory results in malignant melanoma, lung cancer, and other tumors. Compared with lung cancer and melanoma, breast cancer is considered a “weakly immunogenic” tumor, and its immunotherapy effect is not sufficient. Therefore, it is necessary to find new genes to improve the immunotherapy of breast cancer. As correlation analysis can provide critical clues for studying *EMC2* functions and mechanisms, the correlation between the expression level of *EMC2* and the level of immune cell infiltration was evaluated. As shown in **Figure 7A**, the expression of *EMC2* in BRCA was related to the infiltration of 17 immune cells, including CD8^+^ T cells, mast cells, natural killer (NK) cells, T helper cells (Th1, Th2, and Th17), and neutrophils.

### Correlation Between *EMC2* Expression and Immune Cell Biomarkers in BRCA

To further explore the role of *EMC2* in tumor immunity, we used the GEPIA database to determine the correlation of *EMC2* expression with immune cell biomarkers in BRCA. As shown in **Table 2**, *EMC2* showed correlation with B-cell biomarkers (CD19 and CD79A), CD8^+^ T cell biomarkers (CD8B), M1 macrophage biomarkers (IRF5), M2 macrophage biomarkers (CD163 and MS4A4A), dendritic cell biomarkers (HLA-DPB1, CD1C, and NRP1), NK cell biomarkers (KIR2DL3, KIR2DL4, and KIR3DL3), mast cell biomarkers (TPSB2 and TPSAB1), Th1 cell markers (TNF-α), Th2 cell markers (GATA3), Th17 cell markers (STAT3), and Tfh cell markers (BCL6). These findings partially indicate a significant correlation between *EMC2* expression and immune cell infiltration.

**Table 2:** Correlation analysis between EMC2 and biomarkers of immune cells in BRCA determined by GEPIA2 database.

Immune cell	Biomarker	R value	*p* value
B cell	CD19	-0.082	6.80E-03**
	CD79A	-0.09	3.10E-03**
CD4^+^ T cell	CD4	0.032	3.00E-01
CD8^+^ T cell	CD8A	0.0034	9.10E-01
	CD8B	-0.06	4.70E-02*
M1 Macrophage	NOS2	0.054	7.80E-02
	IRF5	0.17	9.40E-09***
	PTGS2	0.022	4.70E-01
M2 Macrophage	CD163	0.13	2.10E-05***
	VSIG4	0.058	5.50E-02
	MS4A4A	0.13	2.20E-05***
Neutrophil	CEACAM8	-0.012	6.90E-01
	ITGAM	-0.055	6.80E-02
	CCR7	-0.0054	8.60E-01
Dendritic cells	HLA-DPB1	-0.17	4.00E-08***
	HLA-DQB1	0.014	6.50E-01
	HLA-DRA	-0.016	6.00E-01
	HLA-DPA1	0.0068	8.20E-01
	CD1C	-0.18	1.60E-09***
	NRP1	0.064	3.40E-02*
	ITGAX	0.027	3.70E-01
NK cells	KIR2DL1	0.029	3.40E-01
	KIR2DL3	0.069	2.30E-02*
	KIR2DL4	0.096	1.60E-03**
	KIR3DL1	0.014	6.50E-01
	KIR3DL2	0.032	3.00E-01
	KIR3DL3	0.076	1.20E-02*
	KIR2DS4	0.048	1.20E-01
Mast cells	TPSB2	-0.16	5.60E-08***
	TPSAB1	-0.18	6.10E-09***
	CPA3	-0.016	6.10E-01
	HDC	-0.046	1.30E-01
	MS4A2	0.015	6.20E-01
Th1	T-bet	-0.043	1.60E-01
	STAT4	0.0091	7.60E-01
	TNF-α	0.093	2.20E-03**
Th2	GATA3	0.087	4.20E-03**
	STAT6	0.036	2.40E-01
	STAT5A	0.028	3.50E-01
	IL13	0.031	3.00E-01
Th17	STAT3	0.25	7.40E-17***
	IL17A	0.043	1.60E-01
Tfh	BCL6	0.068	2.50E-02*

*p value < 0.05; **p value < 0.01; ***p value < 0.001.

### Relationship Between *EMC2* and Immune Checkpoints

PD1/PD-L1, CTLA-4, and TIM-3 are critical immune checkpoints responsible for tumor immune escape. Considering the potential carcinogenic effects of *EMC2* in BRCA, the relationships between BRCA and PD1, PD-L1, CTLA-4, and TIM-3 were evaluated. **Figures 7B–E** shows that *EMC2* expression is significantly positively correlated with PD-L1, CTLA-4, and TIM-3 in BRCA, regulated by purity. From the expression correlation analysis, we also found that *EMC2* was significantly positively correlated with PD-L1, CTLA-4, and TIM-3 in BRCA (**Figures 7F–I**). These results indicate that tumor immune escape may involve the *EMC2*-related carcinogenesis of BRCA.

### *EMC2* Analysis Using RT-qPCR and Human Protein Atlas

**Figure 8:**
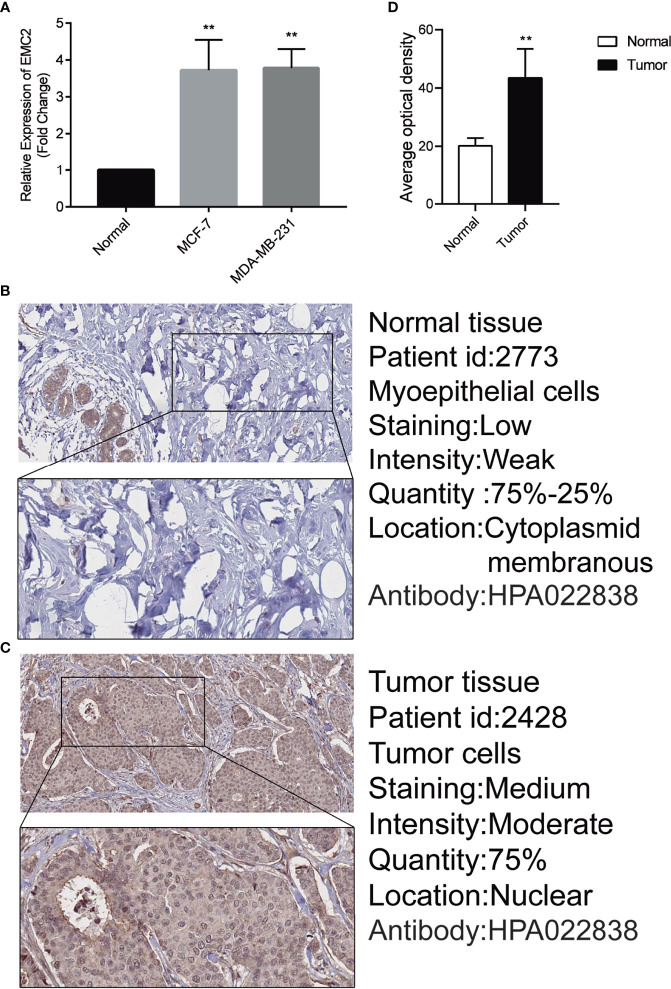
Expression of EMC2 in breast cancer cells and tissues. **(A)** Expression analysis of EMC2 in breast normal and tumor cells. **(B–D)** Expression analysis of EMC2 in breast normal and tumor tissues. **p < 0.01.

After analyzing *EMC2* expression with RT-qPCR (**Figure 8A**) and immunohistochemistry (**Figures 8B–D**), we confirmed that it was upregulated in MCF7 and MDA-MB-231 cells compared to MCF10A cells. The HPA provides a map showing the distribution and relative abundance of proteins in normal human breast tissues and cancer tissues. The protein levels of the *EMC2* was significantly higher in tumor tissues compared with normal tissues based on the HPA database; the EMC2 protein levels were consistent with the RT-qPCR results. The results were significant (*p* < 0.05).

## Discussion

The incidence and mortality of breast cancer in women rank first and second globally, respectively (21). This disease seriously endangers women’s lives and health, and its global burden exceeds that of most other cancers (22). Clarifying the molecular mechanisms of breast cancer can help in the development of effective therapeutic drugs or the search for key clues of promising prognostic biomarkers. An increasing number of studies have shown that the EMC plays a vital role in the initiation and progression of cancer in humans (8, 23). However, in breast cancer, the function of *EMC2* is still unclear, and further research is needed.

Owing to the lack of research on *EMC2* in cancer, in this study, we aimed to perform a pan-cancer analysis of *EMC2* expression using TCGA. We found that *EMC2* is highly expressed in many tumors, but lowly in LAML, OV, TGCT, and THCA. The survival analysis of *EMC2* in tumors shows that BRCA patients with high *EMC2* expression could have a poor survival prognosis. In general, hypermethylation of genes lead to downregulated gene expression (24). However, we found that although *EMC2* is hypermethylated, it is highly expressed in COAD and LUSC. CpG methylation in the promoter region promotes gene transcription in tumors (25). Several potential molecular mechanisms by which gene activation may be regulated under the influence of a hypermethylated gene promoter. First, under certain circumstances, promoter DNA hypermethylation hinders the binding of transcription inhibitors, thereby promoting downstream gene transcription. Second, hypermethylated promoters interact with enhancers but not with silencers to recruit transcription activators and enhance downstream gene transcription. Third, there are multiple variable promoters in certain mammalian genes, which promote the formation of alternative splicing bodies and cause functional differences in the form of protein isoforms. A hypermethylated promoter may induce transcription of the first promoter, increasing the target gene transcript activity and reducing the alternative spliceosome content. However, the specific reasons for the hypermethylation of the promoter region and the high expression of *EMC2* in COAD and LUSC have not been reported. Therefore, the methylation regulation of this gene requires further research.

The cBioPortal database was used to analyze the effect of *EMC2* mutation on tumor prognosis. Although this mutation had no significant effect on the prognosis of breast cancer, it had a significant difference on prostate cancer. *EMC2* mainly uses gene amplification as the main mutation mode, which leads to its high expression in most tumors. In breast cancer, we find the amplification rate of *EMC2* is 11% and the deep deletion rate is 0.1%. Gene amplification can lead to a selective increase in the copy number of certain genes in cells, which is the main mechanism of oncogene activation. This oncogene amplification can lead to the overexpression of the corresponding product, thereby promoting tumor growth and giving cells the potential for immortal proliferation (26). In addition, gene amplification is one of the main mechanisms for tumor cells to evade the efficacy of chemotherapy drugs (27). In this study, we analyzed the relationship between *EMC2* expression and the co-expression of common oncogenes and drug resistance genes in tumors. The results showed that *EMC2* has a significant co-expression relationship with multiple oncogenes and drug resistance genes. The high expression of *EMC2* caused by its own gene amplification may also affect the expression of other genes, thereby improving the proliferation and drug resistance of tumor cells. Therefore, the use of *EMC2* as a target is of great significance to the diagnosis, prognosis, and treatment of tumors. The results of our multivariate Cox regression analysis suggested that *EMC2* can be used as an independent prognostic factor for breast cancer. All of these results collectively indicate that *EMC2* has an important function in breast cancer and could become a new tumor prognostic biomarker.

For decades, the focus of cancer biology research has been the involvement of protein-coding genes. Only recently was a whole class of molecules, called “ncRNAs” was discovered in this regard. ncRNAs play a key regulatory role in shaping cell activity, including carcinogenic molecules and molecules that act in a tumor-suppressing manner. Previous research suggests that most of these miRNAs are tumor suppressor miRNAs in BRCA (28). For example, NEAT1 induced breast cancer progression by regulating the miR-410-3p/*CCND1* axis, indicating that NEAT1 may be a potential therapeutic target in breast cancer (29).

In contrast, the inhibition of miR-410-3p in MCF7 cells resulted in higher proliferation, as assessed using the MTT, plate colony formation, and EdU assays. In addition, miR-410-3p is expressed at low levels with snail in breast cancer samples (30). Differentiation antagonizing non-protein-coding RNA (DANCR) targets and regulates miR-758-3p, and its overexpression can attenuate the anti-cancer effect of miR-758-3p on breast cancer cells (31). The rescue of miR-299-5p expression inhibits breast cancer cell migration and invasion, while the inhibition of miR-299-5p promotes cell migration and invasion (32). Through correlation, expression, and survival analyses, miR-410-3p was found to be the most significant miRNA tumor suppressor upstream of *EMC2*.

According to the ceRNA hypothesis, the lncRNA upstream of the miRNA-410-3p/*EMC2* axis should also be an oncogene in BRCA. Accordingly, we predicted lncRNAs on the miRNA-410-3p/*EMC2* axis and found 55 potential lncRNAs. Through expression, survival, and correlation analyses, we found that LINC00665 was the most significantly upregulated lncRNA. LINC00665 is involved in tumor proliferation, invasion, drug resistance, angiogenesis, and epithelial-mesenchymal transition (EMT) as an oncogene in various tumor, including breast cancer. For example, knockdown of LINC00665 significantly inhibits the growth of breast cancer cells *in vitro*, significantly weakens the migration and invasion abilities, reduces the expression of EMT-related genes, and promotes apoptosis. By contrast, the overexpression of LINC00665 can promotes breast cancer progression (11, 33).

The tumor development process involves mutual transformation and mutual checks and balances between the tumor and immune cells. Recent studies have described the rich tumor immune microenvironment in breast cancer subgroups, these immune infiltrations include innate and adaptive immunity cells, which can be detected and characterized in biopsy specimens and have prognostic value (34). We found that *EMC2* expression was significantly correlated with a variety of immune cells. In addition, *EMC2* was also significantly associated with biomarkers of these infiltrated immune cells. This indicates that tumor immune infiltration may partly account for *EMC2*-mediated carcinogenesis in BRCA.

In addition, immune checkpoint inhibitors are effective in many types of solid tumors. Tumor immune checkpoint blockade immunotherapy targeting PD-1 or CTLA-4 prolongs the OS rate of cancer patients (35). Therefore, we also evaluated the relationship between *EMC2* and immune checkpoints. The results show that the upregulation of *EMC2* expression is significantly related to CD274, CTLA4, and TIM-3 in BRCA, indicating that targeting *EMC2* may improve immunotherapy in BRCA.

**Figure 9:**
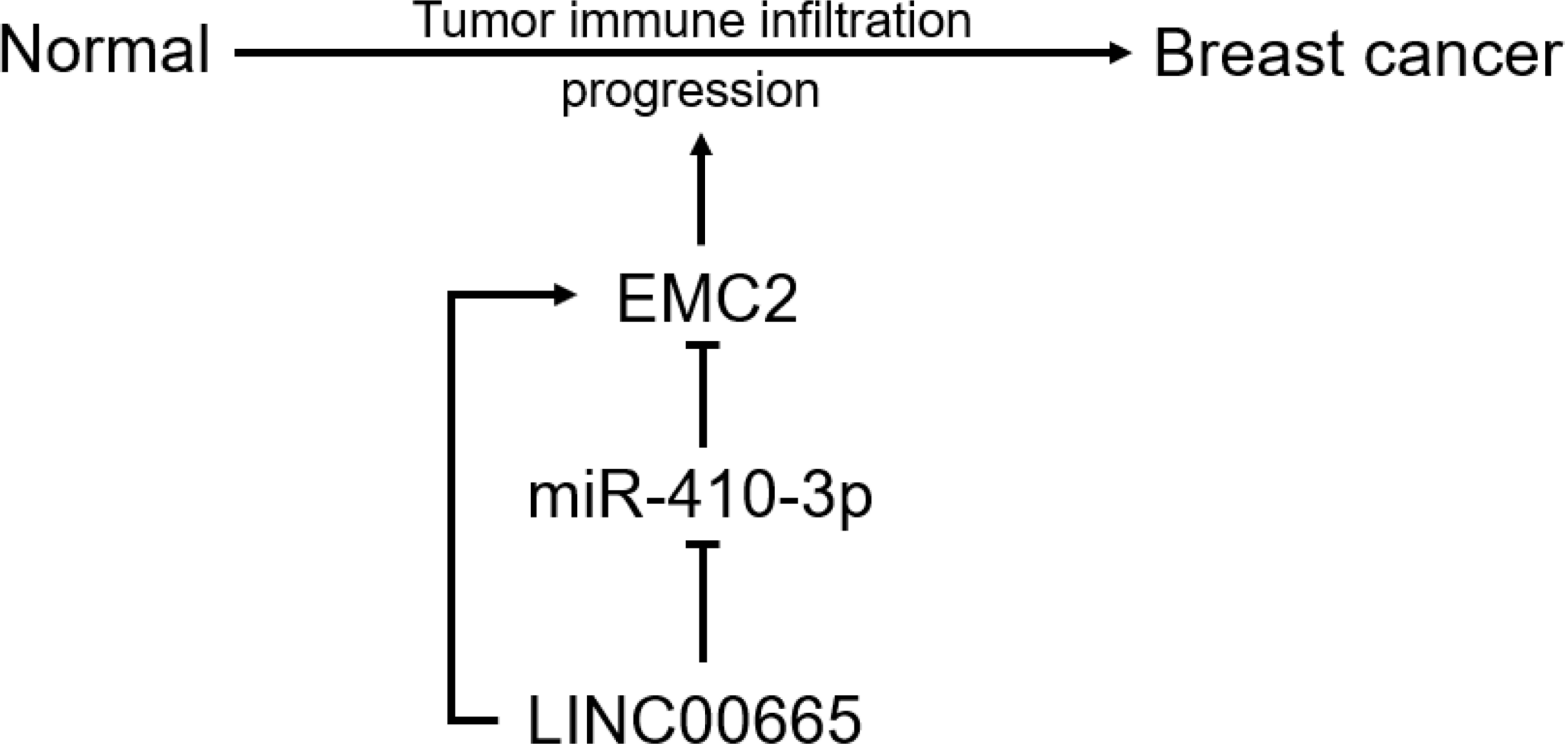
The model of LINC00665-niR-410-3p-*EMC2* axis in carcinogenesis of BRCA.

In conclusion, we clarified that *EMC2* is highly expressed in multiple cancer types, including BRCA, and is positively correlated with unfavorable prognoses in BRCA. We determined the upstream regulatory mechanism of *EMC2* in BRCA, namely the LINC00665/miR-410-3p/*EMC2* axis (**Figure 9**). Our findings also indicate that *EMC2* may exerts its carcinogenic effects by increasing tumor immune cell infiltration and immune checkpoint expression. However, these results need to be validated using basic *in vitro* and *in vivo* experiments and large-scale clinical trials in the future.

## Data Availability Statement

The datasets presented in this study can be found in online repositories. The names of the repository/repositories and accession number(s) can be found in the article/**Supplementary Material**.

## Author Contributions

XL designed the research framework and revised the manuscript. XL, PY, QZ, and LH contributed to the data analysis. XL wrote the manuscript. XS and QQ provided comments during the writing. All authors contributed to the article and approved the submitted version.

## Funding

This study was supported by the National Natural Science Foundation of China (NSFC, NO.82174093).

## Conflict of Interest

The authors declare that the research was conducted in the absence of any commercial or financial relationships that could be construed as a potential conflict of interest.

## Publisher’s Note

All claims expressed in this article are solely those of the authors and do not necessarily represent those of their affiliated organizations, or those of the publisher, the editors and the reviewers. Any product that may be evaluated in this article, or claim that may be made by its manufacturer, is not guaranteed or endorsed by the publisher.
