# Supplementary figures and images for: The ncRNA-Mediated Overexpression of Ferroptosis-Related Gene EMC2 Correlates With Poor Prognosis and Tumor Immune Infiltration in Breast Cancer
Source: Front Oncol. 2021 Dec 8;11:777037. doi: 10.3389/fonc.2021.777037 (PMC8692298; doi:10.3389/fonc.2021.777037)

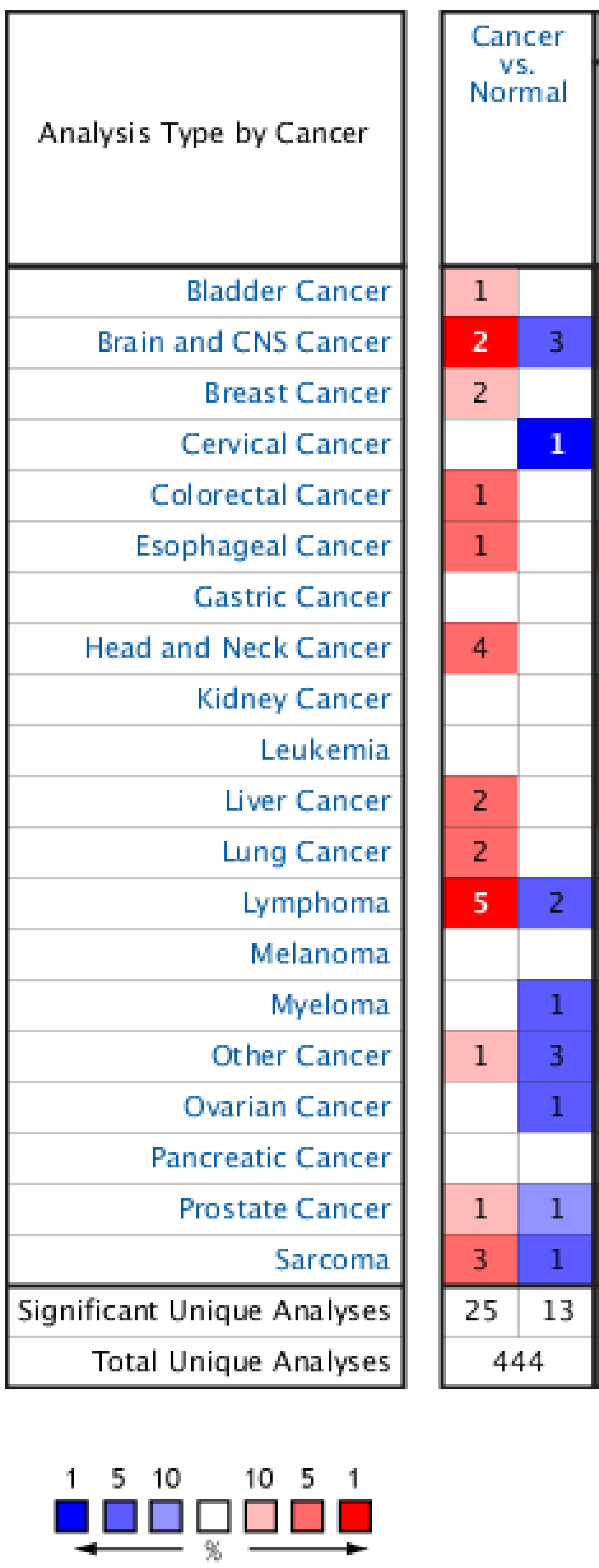

Supplement: Supplementary file 1 [file Image_1.tif]

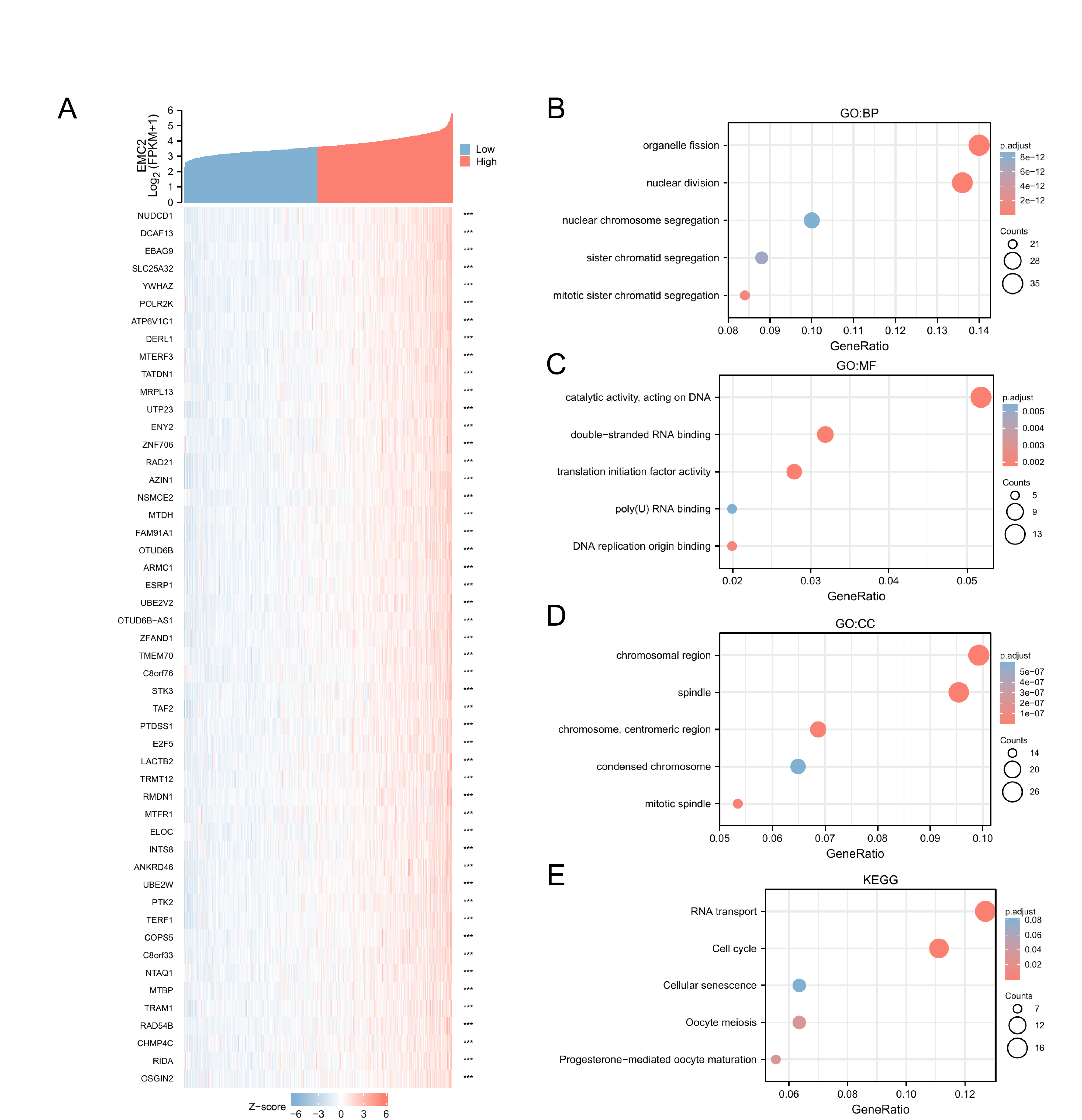

Supplement: Supplementary file 2 [file Image_2.tiff]

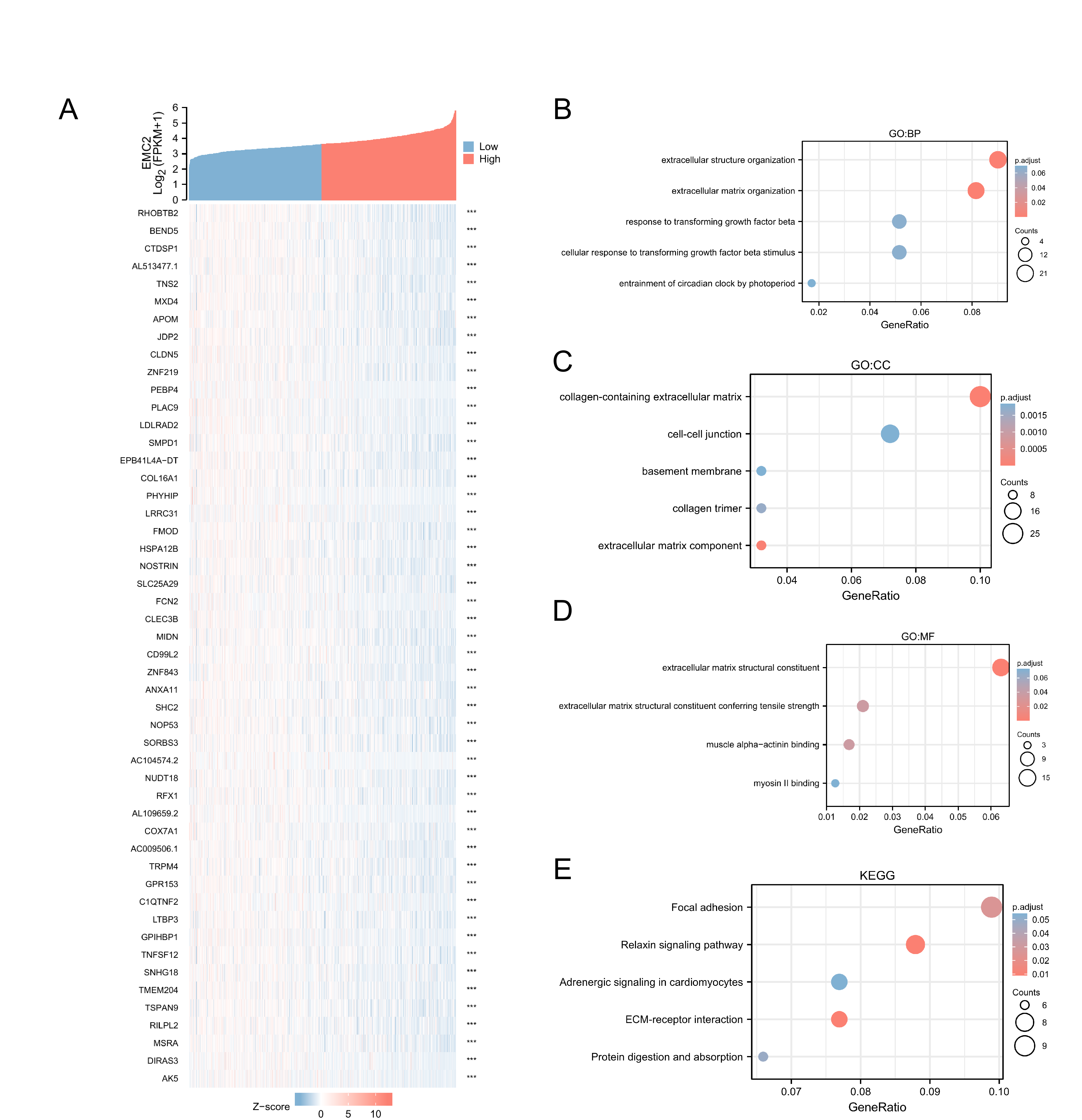

Supplement: Supplementary file 3 [file Image_3.tiff]

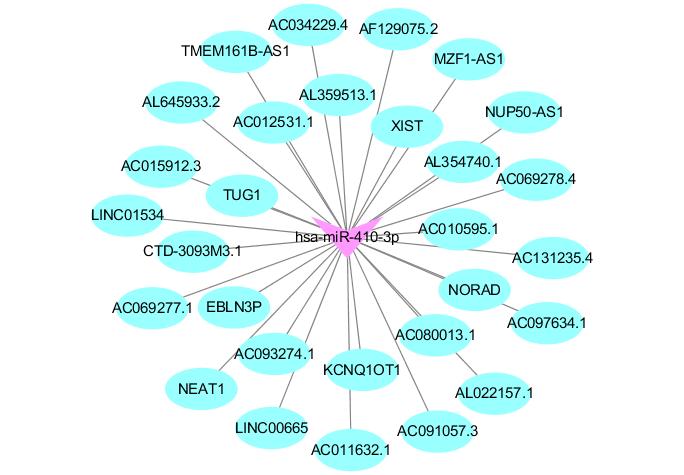

Supplement: Supplementary file 4 [file Image_4.jpeg]
