# Supplementary material for: The ncRNA-Mediated Overexpression of Ferroptosis-Related Gene EMC2 Correlates With Poor Prognosis and Tumor Immune Infiltration in Breast Cancer
Source: Front Oncol. 2021 Dec 8;11:777037. doi: 10.3389/fonc.2021.777037 (PMC8692298; doi:10.3389/fonc.2021.777037)
Supplement: Supplementary file 5 [file Table_1.docx]

Supplementary Table 1 qPCR primers for the study.

| gene | 5’-3’ |
| --- | --- |
| EMC2 | F: CCCCCTTTATGTGTATCTAGTTTGG |
|  | R: TCGCTTGACTCTGTGACTGC |
| GAPDH | F: GGAGCGAGATCCCTCCAAAAT |
|  | R: GGCTGTTGTCATACTTCTCATGG |
