# Supplementary material for: The ncRNA-Mediated Overexpression of Ferroptosis-Related Gene EMC2 Correlates With Poor Prognosis and Tumor Immune Infiltration in Breast Cancer
Source: Front Oncol. 2021 Dec 8;11:777037. doi: 10.3389/fonc.2021.777037 (PMC8692298; doi:10.3389/fonc.2021.777037)
Supplement: Supplementary file 6 [file Table_2.docx]

| **Characteristics** | **Total(N)** | **Univariate analysis** | |  | **Multivariate analysis** | |
| --- | --- | --- | --- | --- | --- | --- |
|  |  | **Hazard ratio (95% CI)** | ***p* value** |  | **Hazard ratio (95% CI)** | ***p* value** |
| T stage | 1079 |  |  |  |  |  |
| T1&T2 | 905 | Reference |  |  |  |  |
| T3&T4 | 174 | 1.608 (1.110-2.329) | **0.012** |  | 1.719 (0.913-3.239) | 0.093 |
| N stage | 1063 |  |  |  |  |  |
| N0 | 514 | Reference |  |  |  |  |
| N1&N2&N3 | 549 | 2.239 (1.567-3.199) | **<0.001** |  | 1.367 (0.755-2.476) | 0.302 |
| Pathologic stage | 1059 |  |  |  |  |  |
| Stage I&Stage II | 799 | Reference |  |  |  |  |
| Stage III&Stage IV | 260 | 2.391 (1.703-3.355) | **<0.001** |  | 1.930 (0.974-3.823) | 0.059 |
| Age | 1082 |  |  |  |  |  |
| <=60 | 601 | Reference |  |  |  |  |
| >60 | 481 | 2.020 (1.465-2.784) | **<0.001** |  | 2.621 (1.644-4.178) | **<0.001** |
| HER2 status | 715 |  |  |  |  |  |
| Negative | 558 | Reference |  |  |  |  |
| Positive | 157 | 1.593 (0.973-2.609) | 0.064 |  | 1.221 (0.719-2.075) | 0.460 |
| EMC2 | 1082 |  |  |  |  |  |
| Low | 541 | Reference |  |  |  |  |
| High | 541 | 1.762 (1.266-2.452) | **<0.001** |  | 1.662 (1.033-2.675) | **0.036** |

**Supplementary Table 2.** Univariate and multivariate Cox analyses in the TCGA cohorts.
